# Experimental induction and mathematical modeling of *Ca*^2+^ dynamics in rat round spermatids

**DOI:** 10.1080/19336950.2020.1826787

**Published:** 2020-10-07

**Authors:** Jonathan Saavedra, Juan G. Reyes, Dino G. Salinas

**Affiliations:** a Instituto de Química, Facultad de Ciencias, Pontificia Universidad Católica de Valparaíso, Valparaíso, Chile; b Centro de Investigación Biomédica, Facultad de Medicina, Universidad Diego Portales, Santiago, Chile

**Keywords:** Calcium dynamics, round spermatid

## Abstract

Cytosolic *Ca^2+^* concentration ([*Ca^2+^*]) has an important role in spermatozoa and hence it regulates fertilization. In male germinal cells, there are indirect evidences that this ion could regulate physiological processes in spermatogenesis. Since little is known about *Ca*^2+^ homeostasis in spermatogenic cells, in this work we propose a mathematical model that accounts for experimental [*Ca^2+^*] dynamics triggered by blockade of the SERCA transport ATPase with thapsigargin in round rat spermatids, without external *Ca^2+^* and with different extracellular lactate concentrations. The model included three homogeneous calcium compartments and *Ca^2+^-ATP*ase activities sensitive and insensitive to thapsigargin, and it adjusted satisfactorily the experimental calcium dynamic data. Moreover, an extended version of the model satisfactorily adjusted the stationary states of calcium modulated by extracellular lactate, which is consistent with the participation of a low affinity lactate transporter and further lactate metabolism in these cells. Further studies and modeling would be necessary to shed some light into the relation between *Ca*^2+^-lactate-ATP homeostasis and cell–cell interactions in the seminiferous tubules that are expected to modulate *Ca*^2+^ dynamics by hormonal factors or energetic substrates in meiotic and postmeiotic spermatogenic cells.

## Introduction

Regulation and dynamics of cytosolic $Ca^{2+}$ concentration $\left({\left[Ca^{2+}\right]}_{cyt}\right)$ constitute one of the key cell signaling events that regulate fertilization, cell proliferation, gene transcription, cell metabolism, cytoskeletal dynamics, and exocytosis, among other cell processes regulated by $Ca^{2+}$ [1–4]. Given the known role of $Ca^{2+}$ in gene expression and protein activity, and the changes in $[Ca^{2+}{]}_{cyt}$ in spermatogenic cells due to different external stimuli [5–8], it can be expected that in male germinal cells $[Ca^{2+}{]}_{cyt}$ could regulate various stages of spermatogenesis. However, little is known about the role of $[Ca^{2+}{]}_{cyt}$ signaling in this process.

Consistent with the idea that $[Ca^{2+}{]}_{cyt}$ changes are important for spermatogenic cell development, rodent spermatogenic cells have the molecular basis needed to respond to extracellular stimuli by changing intracellular $Ca^{2+}$ signals [5,9–15]. Furthermore, these cells express many of the downstream molecular elements that can relay the information of $[Ca^{2+}{]}_{cyt}$ changes to the genome and elicit physiological effects (e.g., calmodulin, calcineurin B; calpains, CAMK, CREM/CREB, NFkB and *Ca*^2+^-activated signaling enzymes) [16–31]. In spite of the physiological and molecular evidence suggesting that $[Ca^{2+}{]}_{cyt}$ homeostasis and signaling machinery are important for normal spermatogenesis, to date, only a few Sertoli cell-derived products have been shown to modify $[Ca^{2+}{]}_{cyt}$ in spermatogenic cells. We have shown that glucose and lactate can modulate $[Ca^{2+}{]}_{cyt}$ in spermatogenic cells, priming spermatids and spermatocytes plasma membrane $Ca^{2+}$ channels for maitotoxin activation [5,7,8]. Additionally, arachidonic acid, a fatty acid released by Sertoli cells can act on spermatogenic cells and release *Ca*^2+^ from intracellular stores [6,32]. Furthermore, several lines of evidence indicate that $[Ca^{2+}{]}_{cyt}$ is important in the spermatogenic process: (a) blockade of ryanodine receptors reduces spermatogonia proliferation and induces meiosis in spermatocytes [10], (b) $Ca^{2+}$ entry regulates the expression of Bcl-xS and Bcl-xL in spermatocytes and spermatids [33], (c) mice deficient in CIB1, a calcium-binding protein, show increased spermatogenic cell apoptosis [34], (d) blockers of voltage-activated calcium channels induce spermatogenesis arrest at the elongating spermatid level in mice [35], and (e) deletion of the enzyme CAMK4 (calcium/calmodulin dependent protein kinase IV) impaired the spermatogenesis [36].

In this work, using the available evidence regarding *Ca*^2+^ homeostatic mechanisms in spermatogenic cells and perturbations in $[Ca^{2+}{]}_{cyt}$ by the blockade of the SERCA transport ATPase with thapsigargin, we developed mathematical models that describe the dynamics of $[Ca^{2+}{]}_{cyt}$ in round spermatids without extracellular $Ca^{2+}$. These models are useful in interpreting and predicting $[Ca^{2+}{]}_{cyt}$ dynamics under physiological conditions or pharmacological interventions.

## Materials and methods

### Animals

Adult (40–60-days old) male Sprague–Dawley rats were acquired from the Animal Facility in the Faculty of Sciences, Universidad de Valparaíso, Chile. The rats were housed in groups of 3–4 animals per cage under a 12 h light:12 h dark cycle with water and rat chow ad libitum. The following procedures were initiated by randomly choosing 1 rat that was euthanized by cervical dislocation following anesthesia with CO_2_. Usually, rats were euthanized in the Animal Facility, and the testes were obtained in a room specially equipped for this procedure.

### Ethical statement

All the experiments were conducted in accordance with the rules established by the Consortium for Development of a Guide for the Care and Use of Agricultural Animals in Agricultural Research and Teaching and by the National Research Council. All experimental protocols were reviewed and approved by the Chilean National Fund for Science and Technology (FONDECYT), and the Ethics Committee of the Pontificia Universidad Católica de Valparaíso (EC-PUCV-10/2013). None of the authors have served in this committee.

### Chemicals

Fura-2 acetoxy methyl ester was purchased from Molecular Probes (Life Technologies, USA). Salts, substrates, thapsigargin and buffers were obtained from Sigma-Aldrich (St. Louis, MO, USA).

### Experimental procedures

#### Rat round spermatid cell isolation

Rat round spermatid cell population was isolated using velocity sedimentation separation in a 2–4% BSA gradient, as described by Romrell et al. [37]. The round spermatid fractions (92 ± 4% purity) were identified both by their size and typical nuclear features after staining with Hoechst 33342 [38]. Most of the measurements in this study were obtained using cell suspensions of round spermatids loaded with specific fluorescent probes, with a Fluoromax 2 fluorimeter (Jobin-Yvon-Spex, NJ, USA).

#### Intracellular $Ca^{2+}$ measurements in rat round spermatids in suspension

Rat round spermatids were suspended (20 × 10^6^ cells/mL) in KH medium (140 mM *NaCl*, 4 mM *KCl*, 1.6 mM *MgCl*_2_, 1.6 mM *KH*_2_*PO*_4_, 10 mM HEPES), pH 7.4, containing or lacking $Ca^{2+}$ (0.5 mM *CaCl*_2_ or 1 mM EGTA, respectively) and having either 5 mM L-Lactate (KH-lactate), or 5 mM glucose (KH-glucose). The cells in KH-lactate were loaded with 5 µM of the calcium probe Fura-2 AM by incubation for 1 h at room temperature under an O_2_ atmosphere, then washed three times at 4°C, and finally re-suspended in the same medium. Fluorescence measurements were performed after adding a concentrated cell suspension (50 µl) to a temperature-regulated and stirred spectrofluorometer cuvette (3.0 mL) that contained 2.55 mL of the four different media described above, giving cell concentrations of 2.94 × 10^6^ cells/mL, to which thapsigargin was added. The $[Ca^{2+}{]}_{cyt}$ determinations were performed using a ratiometric method as described by 39,(39]. Fura-2 calibration was performed by cell lysis with digitonin (20–25 µg/mL) and further addition of 1 mM EGTA to determine Fmin, and addition of 3 mM CaCl_2_ to the digitonin-treated cell suspension to determine Fmax. All measurements were performed without adding external *Ca*^2+^ and 1 mM EGTA, unless indicated otherwise.

## Results

### Effects of thapsigargin on lactate-induced $[Ca^{2+}{]}_{cyt}$

**Figure 1.**
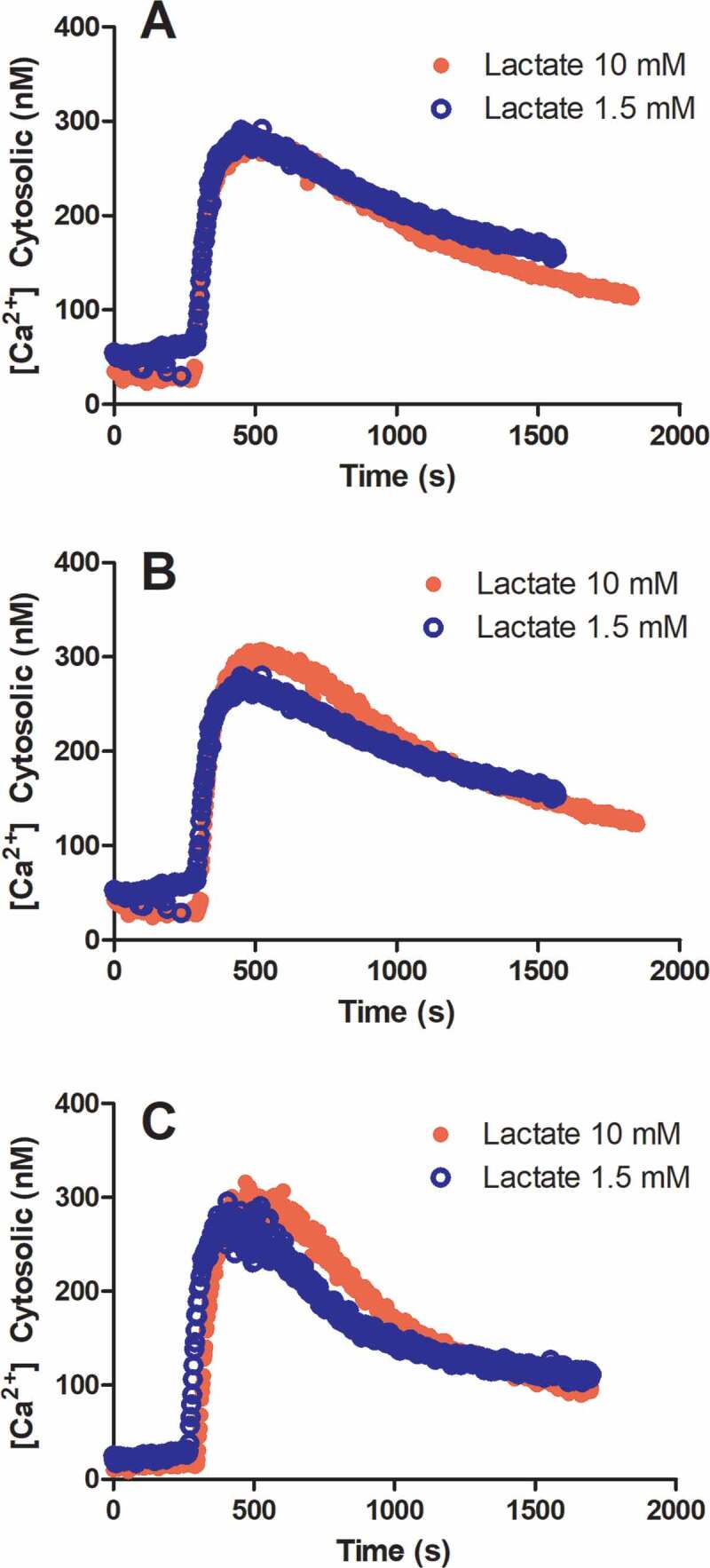
Dynamics of round spermatid $[Ca^{2+}{]}_{ct}$ before and after thapsigargin addition with 1.5 and 10 mM extracellular lactate.

**Figure 2.**
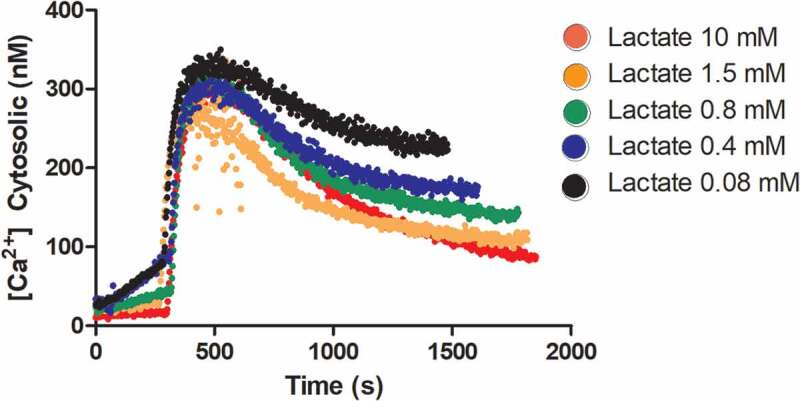
Dynamics of round spermatid cytosolic calcium concentration before and after thapsigargin addition with different extracellular lactate concentrations.

Cells were treated with two lactate concentrations known to induce different basal $[Ca^{2+}{]}_{cyt}$ levels [5], for 0, 5, and 10 min, after which thapsigargin (300 nM) was added. The dynamics of $[Ca^{2+}{]}_{cyt}$ estimated using fura-2 measurements are shown in Figure 1, and these are similar under different conditions. However, there is a slight tendency for $[Ca^{2+}{]}_{cyt}$ to be higher at longer times of lactate incubation before thapsigargin addition. Figure 2 shows measurements of $[Ca^{2+}{]}_{cyt}$ for greater range of extracellular lactate concentrations. Because the steady-state condition was not reached in these measurements, the steady-state $[Ca^{2+}{]}_{cyt}$ value was obtained by extrapolation using exponential decay.

The cells were maintained in KH without lactate until added to a cuvette containing lactate and 0.5 mM EGTA. (a) the recording started immediately after cell addition, (b) The cells were incubated with lactate for 5 minutes before recording, and (c) The cells were incubated with lactate for 10 minutes before recording.

The cells were maintained in KH medium without lactate until were added at −5 min to a cuvette containing KH-lactate and 0.5 mM EGTA.

Table 1 shows a decreasing relationship between extracellular lactate concentration ([*L*]) and $[Ca^{2+}{]}_{cyt}$ before thapsigargin addition and at the extrapolated steady-state $[Ca^{2+}{]}_{cyt}$ after thapsigargin addition. The data were obtained from triplicate experiments similar to Figure 2.

**Table 1.** Extracellular lactate concentrations [*L*] and the corresponding $[Ca^{2+}{]}_{cyt}$ in two cases: immediately before adding thapsigargin and at the estimated steady-state $[Ca^{2+}{]}_{cyt}$ by exponential fitting after adding thapsigargin. (*n* = 3).

[*L*] [mM]	$[Ca^{2+}{]}_{cyt}$ immediately before adding thapsigargin [nM]	Steady-state $[Ca^{2+}{]}_{cyt}$ of the system with thapsigargin [nM]
0.08	50 ± 3.0	240 ± 39
0.4	39 ± 6.9	183 ± 37
0.8	35 ± 7.8	157 ± 20
1.5	24 ± 0.6	103 ± 9
10	14 ± 1.1	61 ± 5

As shown by Herrera et al. [5], $[Ca^{2+}{]}_{cyt}$ was dependent on [*L*] in round spermatids. The dependence of the post-thapsigargin steady-state $[Ca^{2+}{]}_{cyt}$ value on [*L*] strongly suggests that homeostatic mechanisms other than SERCA-ATPases operate following lactate (and its metabolism [5]) treatments of these cells. As will be shown further in this article, these results can be used to propose a mathematical model linking energy metabolism and $[Ca^{2+}{]}_{cyt}$ homeostasis.

### Mathematical models to describe thapsigargin-induced $[Ca^{2+}{]}_{cyt}$ dynamics and lactate-modulated steady-states $[Ca^{2+}{]}_{cyt}$ in round spermatids

**Figure 3.**
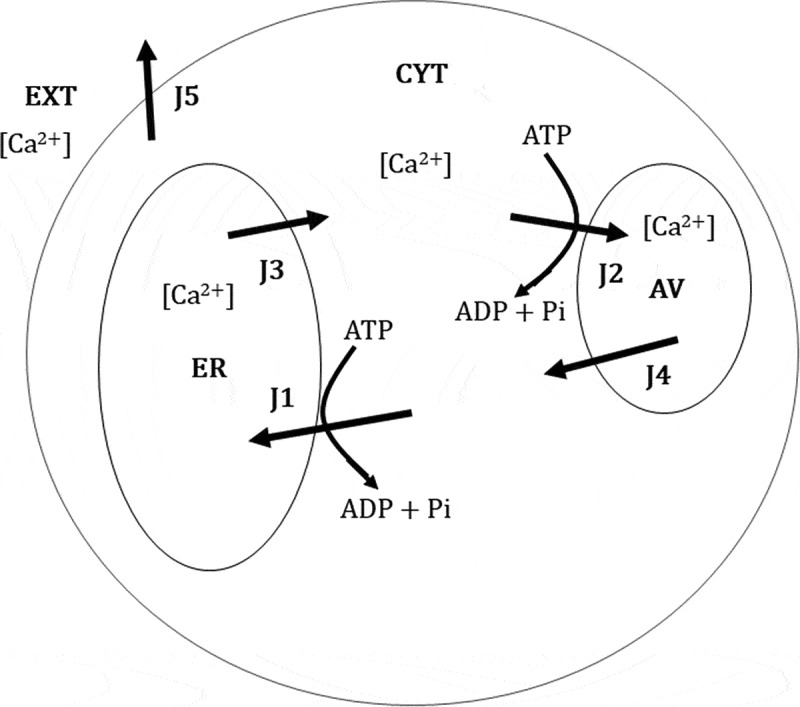
A scheme of Ca^2+^ compartments and fluxes in the absence of external Ca^2+^ in rat round spermatids.

ER, endoplasmic reticulum; CYT, cytoplasm; AV, acidic vesicles. The arrows indicate the compartment toward a positive flux is directed: J1, flux due to the Ca2+-ATPase 1; J2, flux due to the Ca2+-ATPase 2; J3, 4 and 5, leak or exchange fluxes.

Figure 3 shows a schematic of a round spermatid composed of three intracellular *Ca*^2+^ distribution compartments: cytoplasm, endoplasmic reticulum (ER), and acidic vesicles (AV). In this Figure the following calcium fluxes are proposed:

*J*_1_: $Ca^{2+}$ entry from the cytoplasm to the ER due to a SERCA type $Ca^{2+}$ ATPase that transports 2 *Ca*^2+^ ions per hydrolyzed *ATP* [40–43].

*J*_2_: $Ca^{2+}$ entry from the cytoplasm to AV due to a *Ca*^2+^-ATPase that transports one $Ca^{2+}$ per *ATP* hydrolyzed [44].

*J*_3_: $Ca^{2+}$ leakage from the ER lumen toward the cytoplasm.

*J*_4_: $Ca^{2+}$ leakage from the lumen AV toward the cytoplasm.

*J*_5:_ $Ca^{2+}$ efflux from the cytoplasm toward the extracellular medium due to $Na^{+}/Ca^{2+}$ exchanger [45].

#### Basal mathematical model for thapsigargin-induced $[Ca^{2+}{]}_{cyt}$ dynamics

We searched for a minimum number of flux components to model the $[Ca^{2+}{]}_{cyt}$ dynamics in round spermatids after thapsigargin addition, and the following were the preliminary assumptions:

1. The total calcium in the cell was assumed constant (e.g [46–51]), that is, the cell behaves kinetically as a closed system for *Ca*^2+^. This is because outside the cell there is very low free $\left[Ca^{2+}\right]$ (estimated [*Ca*^2+^]*_ext_* = 5 nM) and the $Ca^{2+}$ efflux from the cell is assumed to be null (*J*_5_ = 0).

2. In the cytosol, with volume Vol_cyt_, there is an *f*_cyt_ ratio of free $Ca^{2+}$to total calcium in the same compartment. Similarly, in the ER, with Vol_ret_, there is an *f*_ret_ ratio of free $Ca^{2+}$ to total calcium in the ER; and in AV, with volume Vol_ves_, there is an *f*_ves_ ratio of free $Ca^{2+}$ to total calcium in AV.

3. *J*_3_ and *J*_4_ are proportional to the electrochemical gradient across the membrane.

4. For *J*_3_, the potential difference is constant [52] and is equal to 0 mV [53].

5. For *J*_4_, it is assumed that the membrane potential of the compartment is not zero and meets the Goldman-Hodgkin-Katz equation [54].

6. The membrane area of the different compartments is considered constant during the experiment [55].

According to the previous definitions of fluxes in the scheme of Figure 3, the net fluxes inside the cytosolic, reticular and vesicular compartments (named $J_{Cacyt}^{Net},\;J_{Caret}^{Net}\;and\;J_{Caves}^{Net},$ respectively) are given by

(1) $$J_{Cacyt}^{Net}=-J_{1}-J_{2}+J_{3}+J_{4}$$

(2) $$J_{Caret}^{Net}=J_{1}-J_{3}$$

(3) $$J_{Caves}^{Net}=J_{2}-J_{4}$$

and its relationship with the derivation of the concentration of $Ca^{2+}$ in each homogeneous compartment is given by [56]

(4) $$J_{Cacyt}^{Net}=\frac{Vol_{cyt}}{f_{cyt}}\frac{d{\left[Ca^{2+}\right]}_{cyt}}{dt}$$

(5) $$J_{Caret}^{Net}=\frac{Vol_{ret}}{f_{ret}}\frac{d{\left[Ca^{2+}\right]}_{cyt}}{dt}$$

(6) $$J_{Caves}^{Net}=\frac{Vol_{ves}}{f_{ves}}\frac{d{\left[Ca^{2+}\right]}_{cyt}}{dt}$$

Since we have hypothesized that the cell does not exchange $Ca^{2+}$ with the extracellular compartment, the total intracellular Ca^2+^ must be conserved as follows:

(7) $$[Ca^{2+}{]}_{total}=[Ca^{2+}{]}_{cyt}\frac{Vol_{cyt}}{f_{cyt}}+[Ca^{2+}{]}_{ret}\frac{Vol_{ret}}{f_{ret}}+[Ca^{2+}{]}_{ves}\frac{Vol_{ves}}{f_{ves}}$$

Additionally, in this part, we assume that thapsigargin has been applied, then $J_{1}=0$. Moreover, from Eqs. 1–7 and A10-A12 (with the mathematical expressions for the fluxes, according to Appendix A), the following relations were obtained

(8) $$\frac{d{\left[Ca^{2+}\right]}_{cyt}}{dt}=p-q[Ca^{2+}{]}_{cyt}+r[Ca^{2+}{]}_{ret}-d[Ca^{2+}{]}_{cyt}^{2}$$

(9) $$\frac{d{\left[Ca^{2+}\right]}_{ret}}{dt}=-g[Ca^{2+}{]}_{ret}+g[Ca^{2+}{]}_{cyt}$$

Such that

(10) $$p=\alpha a_{total}P_{Leak2}A_{ves}\frac{f_{cyt}}{Vol_{cyt}}\frac{f_{ves}}{Vol_{ves}}$$

(11) $$q=\alpha P_{Leak2}A_{ves}(\beta \frac{f_{ves}}{Vol_{ves}}+\frac{f_{cyt}}{Vol_{cyt}})+P_{Leak1}A_{ret}\frac{f_{cyt}}{Vol_{cyt}}$$

(12) $$r=P_{Leak1}A_{ret}\frac{f_{cyt}}{Vol_{cyt}}-\alpha \beta P_{Leak2}A_{ves}\frac{Vol_{ret}}{f_{ret}}\frac{f_{ves}}{Vol_{ves}}\frac{f_{cyt}}{Vol_{cyt}}$$

(13) $$d=\frac{f_{cyt}}{Vol_{cyt}}{k_{3}}^{\prime }$$

(14) $$g=\frac{A_{ret}f_{ret}}{Vol_{ret}}$$

#### Basal mathematical model was adjusted to the experimental data by applying 0.3 μM of thapsigargin to cells after 5 min of incubation with 10 mM lactate

**Figure 4.**
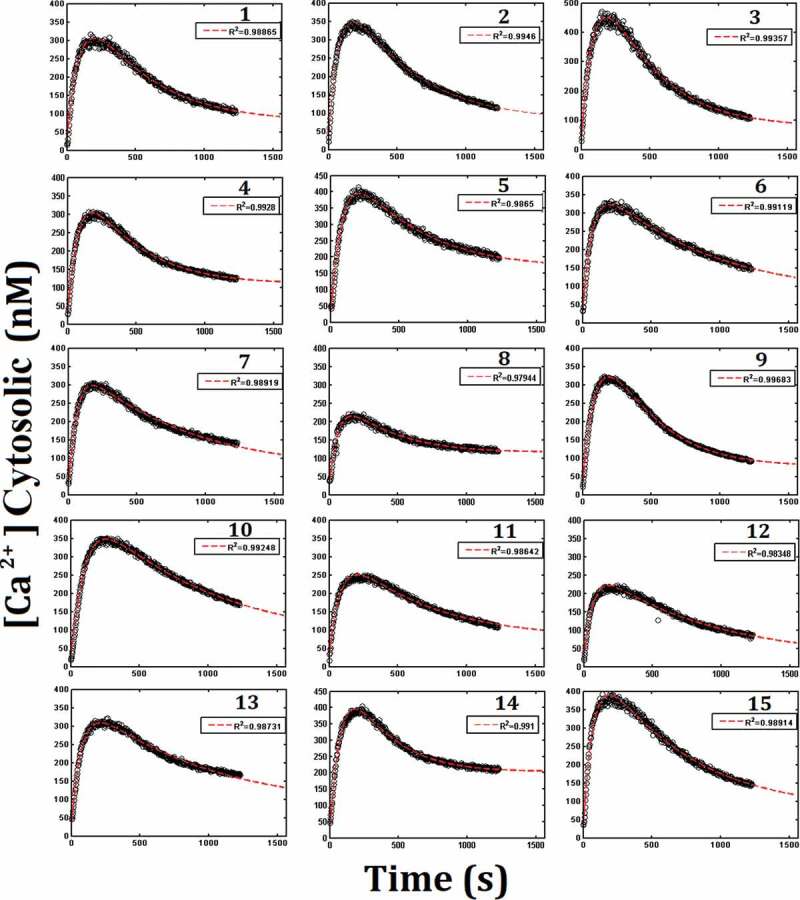
Mathematical model adjustment to the cytosolic calcium concentration data after addition of thapsigargin.

Before adding thapsigargin, $[Ca^{2+}{]}_{cyt}$ values in rat round spermatids were recorded in the ranger of 15 to 50 nM. After 5 min of incubation, 300 nM thapsigargin was added, causing the rapid rise of $[Ca^{2+}{]}_{cyt}$ to a maximum between 300 and 500 nM, and subsequently decreased in slow, exponential, and asymptotic mode, reaching values between 100 and 200 nM. However, these stationary values were not reached during the experiments and, instead, were estimated theoretically by an exponential decay extrapolation. Figure 4 shows the results of 15 different experiments, including all the theoretical fit by means of the differential equation model, as described below.

Round spermatids were maintained in KH-no Ca^2+^-10 mM lactate and 0.5 mM EGTA for 5 minutes before thapsigargin addition. Each record represents an experiment performed on a different rat (*n* = 1 per record). Records are numbered from 1 to 15. The mathematical adjustment to the experimental data was obtained using the model described in the text. In dark circles are shown the experimental data and the discontinuous red line is the mathematical adjustment to the model. (See also Table 2 and 3 for values of the adjustment parameters in the mathematical model).

Using the function ode45 from the MATLAB software, the theoretical values of $[Ca^{2+}{]}_{cyt}$ were obtained by the fifth order Runge-Kutta method. The values of the adjustment parameters for each experiment (Table 2) were obtained using MATLAB’s function fminsearch, which applies the Nelder-Mead method [57,58], to minimize the mean squared error given by the differences between the theoretical and experimental values $[Ca^{2+}{]}_{cyt}$for the total recording after adding thapsigargin. Table 3 shows the statistical analysis of the obtained parameter values, considering dispersions, averages, and the initial parameter values used to start the adjustment. Initial parameters were calculated from the corresponding definition equations using the parameters listed in Table 4.

**Table 2.** Estimated parameters obtained from the mathematical model adjustment. The first column corresponds to each experiment shown in Figure 4, giving 15 sets of adjustment parameters.

Experiment	*p* [s^−1^]	*q* [s^−1^]	*r* [s^−1^]	*d* [s^−1^ nM^−1^]	*g* [s^−1^]	**R^2^**
**1**	9.30x10^−2^	2.13x10^−8^	5.93x10^−5^	2.09x10^−5^	3.40 x10^−3^	0.98
**2**	0.147	6.56x10^−9^	7.56x10^−5^	2.17x10^−5^	3.50x10^−3^	0.99
**3**	6.70x10^−2^	8.87x10^−9^	1.02x10^−4^	1.17x10^−5^	5.70x10^−3^	0.98
**4**	0.267	2.03x10^−8^	6.00x10^−5^	2.22x10^−5^	4.00x10^−3^	0.99
**5**	0.47	2.69x10^−9^	6.52x10^−5^	1.67x10^−5^	2.90x10^−3^	0.98
**6**	0.166	1.48x10^−8^	6.53x10^−5^	2.73x10^−5^	2.00x10^−3^	0.99
**7**	0.366	1.98x10^−9^	5.82x10^−5^	2.68x10^−5^	2.90x10^−3^	0.99
**8**	0.543	2.06x10^−8^	3.96x10^−5^	4.08x10^−5^	3.60x10^−3^	0.97
**9**	5.93x10^−2^	1.32x10^−8^	6.55x10^−5^	1.50x10^−5^	5.30x10^−3^	0.99
**10**	0.2596	7.32x10^−8^	4,70x10^−5^	1.18x10^−5^	3.30x10^−3^	0.98
**11**	6.32x10^−2^	4.34x10^−8^	4.94x10^−5^	3.37x10^−5^	2.00x10^−3^	0.98
**12**	3.25x10^−2^	3.51x10^−9^	4.56x10^−5^	3.76x10^−5^	2.30x10^−3^	0.98
**13**	0.435	1.74x10^−8^	5.25x10^−5^	2.32x10^−5^	2.60x10^−3^	0.98
**14**	0.658	1.66x10^−8^	7.25x10^−5^	1.61x10^−5^	4.70x10^−3^	0.99
**15**	7.23x10^−2^	5.55x10^−10^	8.34x10^−5^	2.14x10^−5^	2.50x10^−3^	0.98

**Table 3.** Analysis of the adjusted parameters shown in Table 2. It is shown the average ($\overset{ˉ}{P})$, the coefficient of variation as percentage (CV%, equal to 100xSD/$\overset{ˉ}{P}$) and the ratio between the average values of the adjusted parameter to the initial estimated value of the parameter ($\overset{ˉ}{P}$/$P_{i}$), for each adjusted parameter.

Parameter	*p* [s^−1^]	*q* [s^−1^]	*r* [s^−1^]	*d* [s^−1^ nM^−1^]	*g* [s^−1^]
Estimated initial value of the parameter ($P_{i}$)	1.68	5.96 x10^−4^	3.93 x10^−4^	2.81 x10^−4^	2.80 x10^−3^
Average of adjusted parameter value ($\overset{ˉ}{P}$)	2.47 x10^−1^	1.77 x10^−8^	6.27 x10^−5^	2.31 x10^−5^	3.38 x10^−3^
CV%	82%	107%	26%	38%	35%
$\overset{ˉ}{P}$/$P_{i}$	1.47 x10^−1^	2.96 x10^−5^	1.60 x10^−1^	8.23 x10^−2^	1.21

**Table 4.** Parameters and variables mentioned in this article and some of their characteristic values. Some values were used only to set the initial conditions of the iterative adjustment methods used in this work.

Parameter or variable	Value and units	Cell type	Reference
J	M s^−1^		
*Vol_cyt_*	8.6x10^−13^ dm^3^	Rat round spermatid	[66]
*Vol_ret_*	1.3 x10^−13^ dm^3^	Rat round spermatid	[66]
*Vol_ves_*	1.8 x10^−15^ dm^3^	Rat round spermatid	[66]
*A_ret_*	3.6 x10^−7^ dm^2^	Rat round spermatid	[66]
*A_ves_*	2.1 x10^−9^ dm^2^	Rat round spermatid	[66]
*Km*	Para ATP = 2 μM	Rabbit skeletal muscle	[71]
*K_3_*	3.36 nmol s^−1^ nM^−2^	Rabbit skeletal muscle	[61]
*K_3_’*	288 nmol s^−1^ nM^−2^	Rabbit skeletal muscle	[61]
*K_4_*	−7.3x10^−13^ dm^3^ s^−1^	Rat uterine smooth muscle	[45]
*K_5_*	9.28x10^−14^ dm^3^s^−1^	Rat uterine smooth muscle	[45]
[*ATP*]	1.33 mM	Rat round spermatid	[62]/Experimental
[*Na^+^*]*_ext_*	142 mM	Rat round spermatid	Experimental
[*Na^+^*]*_int_*	45 mM	Rat round spermatid	Experimental
[*Ca^2+^*]*_ret_*	60 μM	Rat gonadotropes	[63]
[*Ca^2+^*]*_ves_*	40 μM	Mouse macrophage	[72]
[*Ca^2+^*]*_cyt_*	Minimum = 15 nM, Maximum = 300 nM y Steady-state = 77 nM	Rat round spermatid	Experimental
$f_{cyt},\;f_{ret}$,$\;f_{ves}$,	0.01	Mouse pancreatic beta cell	[73]
*P_leak_*	10^−7^ dm s^−1^		A suitable value (Although greater than 10^−8^ dm s^−1^ in [74])

#### Modeling lactate-modulated steady-states $[Ca^{2+}{]}_{cyt}$

The following is a proposed model for the time change of the cytoplasm [*ATP*]:

(15) $$\frac{d\left[ATP\right]}{dt}=\nu +f\left(\left[L\right]\right)-k\left[ATP\right]$$

where $\left[L\right]$ is considered constant, ν a constitutive ATP synthesis rate independent of $\left[L\right]$; f an ATP synthesis function dependent on $\left[L\right]$; and *k*, a first-order constant of ATP hydrolysis, a simplified way of representing the influence of several ATP-hydrolyzing cell processes. The function *f* was associated with lactate transport as a limiting factor of the metabolic flux of lactate, and the transport activity was described by a rectangular hyperbola including an affinity constant $K_{L}$:

(16) $$f\left(\left[L\right]\right)=\frac{\gamma \left[L\right]}{K_{L}+\left[L\right]}$$

$[Ca^{2+}{]}_{cyt}$ **calculations at steady-state as a function of** $\left[L\right]$ **considering a hypothetical net Ca^2+^-ATPase activity**

Assuming a steady-state for **[*ATP*]**,

(17) $$\frac{d\left[ATP\right]}{dt}=0$$

From Eqs. 15–17, it was obtained

(18) $$\left[ATP\right]=\frac{\nu }{k}+\frac{\gamma }{k}\frac{\left[L\right]}{K_{L}+\left[L\right]}$$

However, assuming a steady-state for $\left[Ca^{2+}\right]cyt$.

(19) $$\frac{d{\left[Ca^{2+}\right]}_{cy}}{t}\;=\frac{d{\left[Ca^{2+}\right]}_{ret}}{dt}\;=0$$

The $[Ca^{2+}{]}_{cyt}$ at steady-state ($[Ca^{2+}{]}_{cyt\_st}$) can be calculated as a function of [*L*] from Eqs. 8–9, 13, 17–19 y A7-A8

(20) $$\delta \frac{\phi +\frac{\omega \left[L\right]}{K_{L}+\left[L\right]}}{1+\phi +\frac{\omega \left[L\right]}{K_{L}+\left[L\right]}}{\left[Ca^{2+}\right]}_{cyt\_st}^{2}+B{\left[Ca^{2+}\right]}_{cyt\_st}-1=$$

With thapsigargin.

and having defined:

(21) $$\delta =\frac{f_{cyt}}{p}\frac{{K_{1}}^{\prime }}{{k_{2}}^{\prime }}[ATP_{asaVes}]$$

(22) $$\phi =\frac{\nu }{kK_{MATP}}$$

(23) $$\omega =\frac{\gamma }{kK_{MATP}}$$

(24) $$B\;=\frac{q-r}{p}$$

In the absence of thapsigargin, besides the AV $Ca^{2+}$-ATPase pump, we have activity of the ER $Ca^{2+}$-ATPase pump (J1 > 0), and similar calculations allow us to obtain an equation such as Eq. 20, but replacing δ with $\delta^{\prime }$:

(25) $$\delta^{\prime }\frac{\phi +\frac{\omega \left[L\right]}{K_{L}+\left[L\right]}}{1+\phi +\frac{\omega \left[L\right]}{K_{L}+\left[L\right]}}{\left[Ca^{2+}\right]}_{cyt\_st}^{2}+B{\left[Ca^{2+}\right]}_{cyt\_st}-1=$$

with

(26) $$\delta^{\prime }=\frac{f_{cyt}}{\;}\left(\frac{{\;}_{1}}{k_{2}}[ATP_{asaRet}]+\frac{{K_{1}}^{\prime }}{{k_{2}}^{\prime }}[ATP_{asaVes}]\right)$$

Without thapsigargin.

Other parameters are defined in Appendix A. In this case, as a state immediately prior to the application of thapsigargin (Figure 2), the assumption of the steady-state may not be adequate, being rather a first approximation. The proposed reason for deviations from noncompliance with the steady-state in the referred case will be discussed later.

For simplicity, the $K_{MATP}$ affinity constants of ATP for ER and AV *Ca*^2+^-ATPases were assumed to be the same. However, both the number of each pump and their maximum rates may be different for each case, which is indicated by the primary and non-primary parameters (Appendix A). The model adjustments to the steady-state, given by Eq. 20 and 25, are shown in Figure 6. The adjustment parameters are shown in Table 5.

**Table 5.** Adjusted parameters for Equation 20 and 26. The adjusting parameters were obtained from a descending-step algorithm from initial values. Without thapsigargin (Figure 5a), the initial values for the adjusting parameters were ${\delta_{0}}^{\prime }$ =$\delta_{0}$ and *Φ*_0_, fixing the other parameters to those found for the adjustment in Figure 5b. With thapsigargin (Figure 5b), the initial values for the adjusting parameters were $\delta_{0}$ =1.67 ×10^−4^ (*d*/*p*), *Φ*=0.3162, $\omega_{0}$=1.4142, $K_{L0}=$2, $B_{0}=$1.2083 x10 ^−4^ ((*q-r*)/*p*).

Adjusted parameters for Figure 5a (without thapsigargin)	δ’ =0.0234	*Φ*=0.0145	ω=1.8251	$K_{L}$=52.4576	B=3.6864 x 10^−6^	*R*^2^ = 0.98
Adjusted parameters for Figure 5b (with thapsigargin)	δ =0.0013	*Φ*=0.0110	ω=1.8251	$K_{L}=$52.4576	B=3.6864 x 10^−6^	*R*^2^ = 0.98

**Figure 5.**
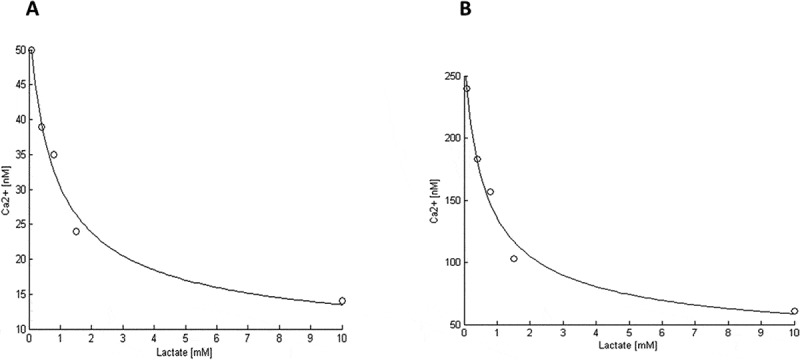
Decreasing relationship between steady-state-cytosolic calcium concentration and extracellular lactate concentration.

(a) Before adding thapsigargin, *R*^2^ = 0.98. (b) After adding thapsigargin, *R*^2^ = 0.98. The data for fitting (o) were taken from Table 1. All the adjusted parameters are shown in Table 5.

So far, the integrated *Ca*^2+^ flux model represented by the dynamic system given by Eq. 8 and 9, was adjusted to the experimental data shown in Figure 4. In its extended version, that is, by considering the functional dependence of $[Ca^{2+}{]}_{cyt}$ on extracellular [*L*], the model was also adjusted to the data shown in Figure 5. Next, we explored the possibility of using alternative models.

#### Other models for adjusting the dynamic and steady-state calcium data

We tested a simpler model that does not consider *Ca*^2+^-ATPases in AV. However, in such a model, the stationary states of $[Ca^{2+}{]}_{cyt}$ in the presence of thapsigargin are independent of ATP, and hence it would be lactate independent, unlike what is observed in the experimental data (Figure 5b)

Similarly, the change of the original closed model into an open model using a $Na^{+}/Ca^{2+}$ exchanger in the plasma membrane (Appendix B) could not be adjusted to the data in Figure 4. In this model, the steady-state $[Ca^{2+}{]}_{cyt}$ after thapsigargin does not depend on [*ATP*] (Eqs. B21 and B22); therefore, it cannot be adjusted to the data points shown in Figure 5b.

## Discussion

In this work, the cytosolic calcium of round rat spermatids was studied by fluorometric methods. Since the measurements were applied to samples composed of many millions of cells, it is possible that the dynamics of calcium per cell are qualitatively different from the average dynamics obtained in these experiments. In any case, the basal calcium records obtained were stationary or slightly increasing. Following the addition of thapsigargin, an abrupt increase and a slow exponential decay toward a steady-state were observed. These records were modeled mathematically considering the structural elements of a likely average model cell. With respect to obtaining parameters to adjust the proposed model to experimental data, it is important to mention that the parameters used at the beginning of all the adjustment algorithms were determined according to bibliographic data, experimental evidence, or simple convenience to obtain biologically plausible parameters (Appendix C).

The proposed mathematical model to reproduce the experimental dynamics of $[Ca^{2+}{]}_{cyt}$ in round rat spermatids considers the calcium fluxes given in Figure 4 and structural elements such as the number of compartments, their homogeneity, their volume and their free calcium fractions, in addition to the parameters of each $Ca^{2+}$ flux. The first $Ca^{2+}$ measurements were made to optimize the recording time and lactate concentrations used. Thapsigargin, a $Ca^{2+}$ -*ATP*ase 1 blocker present in the ER, was used to perturb the cytosolic calcium and test the relevance of the mathematical model.

In the experiments where thapsigargin was added to cells incubated with lactate, different records of $[Ca^{2+}{]}_{cyt}$ versus time were obtained. However, despite being different cell samples, in all data experiment the same set of parameter values was used to initialize the adjustment algorithm. Finally, after parameter adjustment, a different set of optimized parameter values was obtained for each experiment. This diversity of parameter settings is consistent with the fact that each experiment was conducted on different samples (Figure 4, Tables 2 and 3). Table 3 shows that the parameters obtained in Figure 4 have standard percentage deviations between 26% and 107%. However, comparing the averages of each obtained parameter values with respect to the initially assumed parameter values (that is, the values to initialize the adjustment algorithm), it was observed that the values of *g* were very similar, while *p, r*, and *d* did not differ by more than one order of magnitude, and *q* differed by 5 orders of magnitude. In the latter case, it could be that the complex composition of *q* by other parameters (Eq. 11) determines a highly amplified global error. Moreover, in Eq. 8 we must consider that *q*[Ca^2+^]_cyt_ is much less than *d*[Ca^2+^]^2^. Therefore, this difference (corresponding to at least 5 orders of magnitude, according to Table 3 and Figure 4) would determine an increased sensitivity of the *q* parameter with respect to measurement and rounding errors. On the contrary, the aforementioned similarity between the adjusted *g* and the assumed initial *g* would agree with the composition of the parameter *g* (Eq. 14) being simpler and that the assumed values of the parameters that compose it are more reliable ($\;f_{ret},A_{ret},Vol_{ret}$). In any case, the proposed model was adequately adjusted to each of the experimental records shown in Figure 4. Although all adjustment parameter values are within acceptable physiological ranges (Table 3), it is possible that for any of the 15 experimental records in Figure 4 the obtained set of parameter values is not unique. Therefore, the current values should be verified in future experiments, such as for single cell recording experiments.

Any suitable mathematical model of the dynamics of $[Ca^{+2}{]}_{cyt}$ should be able to explain the dependence of stationary calcium states on lactate (Statistical adjustment in Figure 3, adjustment of the model proposed in Figure 6). In that sense, a simpler model, without $Ca^{2+}$ -*ATP*ase, does not account for such stationary states with thapsigargin, nor a more complex model, with $Na^{+}/Ca^{2+}$ exchanger activity. Instead, using the model originally proposed in this work, we observed that by replacing some parameters by expressing $\left[ATP\right]$ as a function of $\left[L\right]$, it was possible to model $[Ca^{2+}{]}_{cyt}$ stationary states before thapsigargin and after its application. It was assumed that the rate of ATP synthesis is given by two terms: a constant term that represents constitutive synthesis of ATP that is independent of lactate (ν) plus one term proportional to a hyperbolic function of extracellular lactate [59]. Furthermore, it was assumed that the overall ATP degradation process is represented as first-order expression. In this extended model, which incorporates $\left[L\right]$ to explain variations of $[Ca^{2+}{]}_{cyt}$ stationary states, it was assumed that ATP affinity constants for both $Ca^{2+}$ -*ATP*ase (1 and 2, ER and AV, respectively) were equal. As a result of the adjustment of this extended model to the data of stationary states (pre- and post-thapsigargin), it was found that the set of adjustment parameters meets the following conditions that validate the model:

- Parameter difference: $\delta^{\prime }>\delta$, which indicates that stationary states are more influenced by $Ca^{2+}$ -*ATP*ase activity in case without thapsigargin than with thapsigargin. In particular, of the values shown in Table 5, we can assume that $Ca^{2+}$ -*ATP*ase activity in the ER membrane is approximately 17 times that of the AV membranes ($\left(\delta^{\prime }-\delta )/\delta =17\right)$.

- Equal parameters: The following parameters are similar in cases of steady-state pre and postthapsigargin (Figure 6 and Table 5): B (includes parameters that determine the dynamics of calcium), ω (includes parameters of transport and lactate metabolism) and $K_{L}$ (Affinity constant of the lactate transporter). Interestingly, a $K_{L}=$53 mM was obtained, a high Km value as reported for the low affinity lactate transporter [59,60]. Regarding $\phi =\frac{\nu }{kK_{MATP}}$, our model requires the same parameter values in the stationary states before and after thapsigargin treatment. However, ϕ diminished 24% after thapsigargin addition. This decrease is interesting, because, it could be due to a hypothetical decrease in ν, that is, a diminished constitutive synthesis of ATP. Moreover, this is consistent with the notorious deviation from the steady-state condition prior to thapsigargin in cases of low $\left[L\right]$, where there is a very low hyperbolic term contribution to the synthesis of ATP.

In conclusion, in the proposed mathematical model for round rat spermatids, consisting of a closed system, three homogeneous compartments, and $Ca^{2+}$ -*ATP*ase activities sensitive and insensitive to thapsigargin, adjust satisfactorily to the experimental calcium dynamics data. In its extended version, which considers elements of energy metabolism, it also adjusts satisfactorily to the stationary states of calcium modulated by lactate, while a spontaneous decrease in constitutive synthesis of ATP would be enough to explain some deviations from the proposed mathematical model. The compartments of the model can be the sum of several compartments of the same type. In another area, the adjustment of the model to the steady-state data modulated by lactate in round rat spermatids suggests the involvement of a low-affinity lactate transporter, which would be a limiting element in the synthesis of ATP for the activity of the $Ca^{2+}$ -*ATP*ase activities AV pumps. Therefore, more research is necessary in this regard. In broader terms, further studies would be useful to clarify important details of lactate transport and ATP homeostasis, so that a more precise mathematical model can be developed to contribute to the study of intracellular calcium modulated by hormonal factors or metabolic energy substrates.

## APPENDIX A

**General mathematical description of** $Ca^{2+}$ **fluxes in a model for rat round spermatids.**

**i) Calculation of** $Ca^{2+}$ **flux from the cytoplasm to the ER by** $Ca^{2+}$ **-*ATP*ase (**$J_{1}$).

$J_{1}$ can be calculated as follows.

(A1) $$J_{1}=2Vol_{cyt}V_{hydATP}$$

where factor 2 represents the transport stoichiometry of 2 $Ca^{2+}$ per *ATP*. $V_{hydATP}$ is the hydrolysis rate of ATP (in this study it is considered volume activity) due to the $Ca^{2+}$ -*ATP*ase pump, such that [61]

(A2) $$V_{hydATP}=V_{hyd}^{max}\frac{\left[ATP\right]}{K_{MATP}+\left[ATP\right]}$$

We assumed a constant $\left[ATP\right]$, in accordance with experimental observations [62]. The maximum hydrolysis rate ($V_{hyd}^{max}$) is given by [61]

(A3) $$V_{hyd}^{max}=\frac{K_{1}[ATP_{asaRET}]}{\left(1+k_{2}/[Ca^{+2}{]}_{cyt}^{2}\right)\left(1+C_{1}[Ca^{+2}{]}_{ret}^{2}\right)}$$

where [*ATP_asaRET_*] is the ATPasa-surface concentration and *K_1_, k_2_* y C_1_ are constants. Here, it was assumed that *C_1_* = 1,4x10^6^ M^−2^ [61] and ${\left[Ca^{2+}\right]}_{ret}$ = 60 μM [63]. Thus, *C_1_*${\left[Ca^{2+}\right]}_{ret}^{2}$= 5 × 10^−3^ ≪ 1. Moreover, *k_2_* ≫ ${\left[Ca^{2+}\right]}_{cyt}^{2}$ since *k_2_* = 6,3x10^6^ nM^2^ [61] and the maximum experimental concentration of cytosolic calcium was less than 500 nM in the records shown in Figure 4. Thus, the following approximation was used:

(A4) $$V_{hyd}^{max}\approx \frac{K_{1}}{k_{2}}[Ca^{2+}{]}_{cyt}^{2}[ATP_{asaRET}]$$

Defining

(A5) $$k_{3}\equiv 2Vol_{cyt}\frac{K_{1}}{k_{2}}[ATP_{asaRet}]\;\frac{\left[ATP\right]}{K_{matp}+\left[ATP\right]}$$

From Eqs. A2, A4-A5 in A1, it is obtained

(A6) $$J_{1}=k_{3}[Ca^{2+}{]}_{cyt}^{2}$$

**ii) Calculation of** $Ca^{2+}$ **flux from the cytoplasm to the AV through a** $Ca^{2+}$ **-*ATP*ase pump (**$J_{2}$).

In this case, the analysis to determine $J_{2}$was similar to that of $J_{1}$, but in this case, 1 mol of ATP hydrolysis was associated with the transport of 1 mol of $Ca^{2+}$ through the $Ca^{2+}$ -*ATP*ase pump [64]. The corresponding kinetic parameters are marked by an apostrophe. Thus, *k*_3ʹ_ is defined as

(A7) $${k_{3}}^{\prime }\equiv Vol_{cyt}\frac{K_{1^{\prime }}}{k_{2^{\prime }}}[ATP_{asaVes}]\;\frac{\left[ATP\right]}{K_{MATP}+\left[ATP\right]}$$

(Similar to $k_{3}$ and using, for simplicity, the same value of $K_{MATP}$)

Therefore, $J_{2}$ is given by

(A8) $$J_{2}={k_{3}}^{\prime }[Ca^{+2}{]}_{cyt}^{2}$$

**iii) Calculation of** $Ca^{2+}$ **leakage from ER (**$J_{3}$).

Assuming that the reticular membrane potential tends to zero [52,53], $J_{3}$is defined only by the concentration gradient of $Ca^{2+}$, a permeability parameter ($P_{Leak1})$and the surface area of the reticulum ($A_{ret}$)

(A9) $$J_{3}=\;A_{ret}P_{Leak1}\left({\left[Ca^{2+}\right]}_{ret}-{\left[Ca^{2+}\right]}_{cyt}\right)$$

**iv) Calculation of** $Ca^{2+}$ **leakage from AV (**$J_{4}$).

Applying the Goldman-Hodgkin-Katz equation to the calculation of the $J_{4}$, we obtained

(A10) $$J_{4}=\;\alpha A_{ves}P_{Leak2}\left(\beta {\left[Ca^{2+}\right]}_{ves}-{\left[Ca^{2+}\right]}_{cyt}\right)$$

With *P_leak2_* the permeability of $Ca^{2+}$ in the AV membrane,

(A11) $$\alpha \equiv \frac{z\frac{F\psi }{RT}}{\beta -1}$$

and

(A12) $$\beta \equiv e^{z\frac{F\psi }{RT}}$$

Such that *z* is the charge of the ion (for $Ca^{2+}$, *z* = 2) and ψ is the vesicular membrane potential.

## APPENDIX B

**Effects of the** $Na^{+}/{C_{a}}^{2+}$ **exchanger on the integrated** $Ca^{2+}$ **flux model**

The following conditions were assumed:

**i) The resting plasma membrane potential remains constant.**

It was assumed that the plasma membrane potential remained at −22 mV. This was because this potential depends highly on $Cl^{-}$ [52], unlike $Na^{+}$ and $Ca^{2+}$, and it has been reported that the cytosolic concentration of $Cl^{-}$ does not vary [65].

**ii) The extracellular** $Ca^{2+}$ **concentration is negligible.**

From the described experimental conditions and using a cellular volume of about 8.6 × 10^−13^ dm^3^ [66], we estimated that the total intracellular volume is less than 0.5% of the extracellular volume. Then, we assumed that the extracellular $Ca^{2+}$ concentration was constant during the experiments, being equal to 2 nM in a medium with 0.5 mM EGTA, that is, a negligible value.

**iii) Extracellular** $Na^{+}$ **and** $Ca^{2+}$ **concentrations remain constant.**

If the experimental system used the $Na^{+}/Ca^{2+}$ exchanger to exchange 3 $Na^{+}$ for 1 $Ca^{2+}$ [67], it should operate according to an extracellular $Na^{+}$ concentration equal to 142 mM and an intracellular $Na^{+}$ concentration of 45 mM [65]. We estimated that the $Ca^{2+}$ outflux necessary to remove the thapsigargin-induced increase in $[Ca^{2+}{]}_{cyt}$ was not sufficient to significantly vary the extracellular and intracellular $Na^{+}$ concentrations. From the experimental conditions described in Material and Methods and using a cellular volume of about 8.6 × 10^−13^ dm^3^ [66] and an internal $Ca^{2+}$ concentration of 500 µM, the calculations allowed us to estimate that for $Na^{+}$ the intracellular concentration increases at most 3.3% and the extracellular concentration does not change.

**iv) The** $Na^{+}/Ca^{2+}$ **exchanger is inactive under the dynamic range of** $Ca^{2+}$

Here, it was assumed that the $Na^{+}/Ca^{2+}$ exchanger was inactive at low concentrations of cytosolic $Ca^{2+}$ [68,69] before the addition of thapsigargin. We assumed that, after adding thapsigargin, cytosolic $Ca^{2+}$ concentrations increase at values close to the half-activation constant of the $Na^{+}/Ca^{2+}$ exchanger (from 25 nM to 600 nM, depending on the species and cell type) [70].

- $Ca^{2+}$ **outflux from the cytoplasm to the extracellular space, due to the** $Na^{+}/Ca^{2+}$ **exchanger (**$J_{5}$)

To calculate $J_{5}$, the following equation was used [45]:

(B1) $$J_{5}=-Vol_{cyt}G_{Na/Ca}\frac{{\left[Ca^{+2}\right]}_{cyt}}{{\left[Ca^{+2}\right]}_{cyt}+K_{Na/Ca}}\left[V_{m}-V_{m,Na/Ca}\right]$$

(The minus sign here is due to differences in the flux sense definition)

Such that *G_Na/Ca_* (=5.7297 × 10^4^ nM s^−1^ V^−1^) is the conductance of the $Na^{+}/Ca^{2+}$ exchanger on the plasma membrane, *K_Na/Ca_* (=7 × 10^3^ nM) is a kinetic parameter, *V_m_* is the membrane potential, and *V_m,Na/Ca_* is the equilibrium potential of the exchanger [45].

(B2) $$V_{m,Na/Ca}=3E_{Na}-1E_{Ca}$$

with *E_Na_* and *E_ca_* the Nernst potentials for $Na^{+}$ and $Ca^{2+}$, respectively, under a 3:1 Na^+^/Ca^2+^ stoichiometry:

(B3) $$E_{Na}=\frac{RT}{F}ln\frac{{\left[Na^{+}\right]}_{ext}}{{\left[Na^{+}\right]}_{int}}$$

(B4) $$E_{Ca}=\frac{RT}{2F}ln\frac{{\left[Ca^{+2}\right]}_{ext}}{{\left[Ca^{+2}\right]}_{cyt}}$$

*R* is the gas constant, *T* is the temperature in Kelvin degrees and *F* is the Faraday constant.

From equations 22 to 25, we obtained

(B5) $$J_{5}=[Ca^{2+}{]}_{cyt}\left[K_{4}-K_{5}ln{\left[Ca^{2+}\right]}_{cyt}\right]$$

Such that

(B6) $$K_{4}=\frac{G_{Na/Ca}}{K_{Na/Ca}}Vol_{cyt}V_{m}-\frac{G_{Na/Ca}}{K_{Na/Ca}}Vol_{cyt}\frac{3RT}{F}ln\frac{{\left[Na^{+}\right]}_{ext}}{{\left[Na^{+}\right]}_{int}}+\frac{G_{Na/Ca}}{K_{Na/Ca}}Vol_{cyt}\frac{RT}{2F}ln[Ca^{2+}{]}_{ext}$$

(B7) $$K_{5}=\frac{G_{Na/Ca}}{K_{Na/Ca}}Vol_{cyt}\frac{RT}{z_{Ca}F}$$

**- Formulation of a system of differential equations to study calcium dynamics**

Considering the Eqs. 1–6, *J*_1_ = 0 and the mathematical expressions for the flows, Eqs A8-A10 and B5, it can be shown that:

$$\frac{d{\left[Ca^{2+}\right]}_{cyt}}{dt}=a[Ca^{2+}{]}_{ves}-b[Ca^{2+}{]}_{cyt}+c([Ca^{2+}{]}_{ret}-[Ca^{2+}{]}_{cyt})$$

(B8) $$-d[Ca^{2+}{]}_{cyt}^{2}+[Ca^{2+}{]}_{cyt}\left(e-fln{\left[Ca^{2+}\right]}_{cyt}\right)$$

(B9) $$\frac{d{\left[Ca^{2+}\right]}_{ret}}{dt}=-g[Ca^{2+}{]}_{ret}+g[Ca^{2+}{]}_{cyt}$$

(B10) $$\frac{d{\left[Ca^{2+}\right]}_{ves}}{dt}=h[Ca^{2+}{]}_{cyt}^{2}-i[Ca^{2+}{]}_{ves}+\frac{ib}{a}[Ca^{2+}{]}_{cyt}$$

Such that

(B11) $$a=\frac{A_{ves}f_{cyt}}{Vol_{cyt}}\alpha P_{Leak2}\beta$$

(B12) $$b=\frac{A_{ves}f_{cyt}}{Vol_{cyt}}\alpha P_{Leak2}$$

(B13) $$c=P_{Leak1}A_{ret}\frac{f_{cyt}}{Vol_{cyt}}$$

(B14) $$d=\frac{f_{cyt}}{Vol_{cyt}}{k_{3}}^{\prime }$$

(B15) $$e\;=\frac{f_{cyt}}{Vol_{cyt}}K_{4}$$

(B16) $$f=\frac{f_{cyt}}{Vol_{cyt}}K_{5}$$

(B17) $$g=\frac{A_{ret}f_{ret}}{Vol_{ret}}P_{Leak1}$$

(B18) $$h=\frac{f_{ves}}{Vol_{ves}}{k_{3}}^{\prime }$$

(B19) $$i=\frac{A_{ves}f_{ves}}{Vol_{ves}}\alpha P_{Leak2}\beta$$

At steady-state, we can define

(B20) $$\frac{d{\left[Ca^{2+}\right]}_{cyt}}{dt}=\frac{d{\left[Ca^{2+}\right]}_{ret}}{dt}\;=\frac{d{\left[Ca^{2+}\right]}_{ves}}{dt}\;=0$$

Then, from Eqs. B8-B20, two possible values for $[Ca^{2+}{]}_{cyt\_st}$ (the steady-states of $[Ca^{2+}{]}_{cyt}$) were derived:

$[Ca^{2+}{]}_{cyt\_st}=0\;$ (B21) and $[Ca^{2+}{]}_{cyt\_st}=e^{\frac{K_{4}}{K_{5}}}$ (B22)

None of these values is *ATP* dependent and then cannot explain the correlation between the steady-state value of $[Ca^{2+}{]}_{cyt}$ and the lactate extracellular concentration. Thus, we can conclude that the Na+/Ca2+ exchanger alone cannot explain the steady-state lacte-dependence of $[Ca^{2+}{]}_{cyt\_st}$
